# Sequential Separation of Essential Oil Components during Hydrodistillation of Fresh Foliage from Azorean *Cryptomeria japonica* (Cupressaceae): Effects on Antibacterial, Antifungal, and Free Radical Scavenging Activities

**DOI:** 10.3390/plants13131729

**Published:** 2024-06-22

**Authors:** Filipe Arruda, Ana Lima, Tanner Wortham, Alexandre Janeiro, Tânia Rodrigues, José Baptista, José S. Rosa, Elisabete Lima

**Affiliations:** 1 Institute of Agricultural and Environmental Research and Technology (IITAA), University of the Azores, 9700-042 Angra do Heroísmo, Portugal; filipe.mp.arruda@uac.pt (F.A.); ana.pr.lima@uac.pt (A.L.); alex-19961917@hotmail.com (A.J.); jose.ab.baptista@uac.pt (J.B.); 2 Department of Biology (DB), Faculty of Science and Technology, University of the Azores, 9500-321 Ponta Delgada, Portugal; tanyamsrod@gmail.com (T.R.); jose.ss.rosa@uac.pt (J.S.R.); 3 Department of Physics, Chemistry and Engineering (DCFQE), Faculty of Science and Technology, University of the Azores, 9500-321 Ponta Delgada, Portugal; 4 The Perfumery, 621 Park East Blvd., New Albany, IN 47150, USA; twortham@theperfumery.com; 5 Biotechnology Centre of Azores (CBA), University of the Azores, 9700-042 Angra do Heroísmo, Portugal

**Keywords:** biomass residue valorization, circular economy, essential oil fractionation, high value-added products, multi-bioactivities, terpenoids

## Abstract

*Cryptomeria japonica* wood industry generates large amounts of foliage biomass residues. Due to the increasing applications and markets for essential oils (EOs), fresh Azorean *C. japonica* foliage (Az–CJF) residues are used for local EO production. Hydrodistillation (HD), a common process for obtaining EOs, also provides the possibility to fractionate them. Thus, this study evaluated the in vitro antimicrobial and antioxidant activities of six Az–CJF EO fractions (Frs. 1–6), collected at sequential HD timeframes (HDTs: 0–2, 2–10, 10–30, 30–60, 60–120, and 120–240 min), in comparison to the crude EO, obtained from a non-fractionated HD (0–240 min HDT). Antimicrobial activities were assessed via disc diffusion method against seven bacteria (foodborne and/or human pathogens) and two *Penicillium* spp. (phytopathogenic fungi), and antioxidant activity was estimated using DPPH and ABTS assays. Concerning the antibacterial activity, all the EO samples were effective only toward Gram-positive bacteria. Fractions 1–3 (<30 min HDT) were the most active, with growth inhibition zones (GIZ) of 7.0–23.3 mm (1.4–2.2 times higher than those of the crude EO), being *Bacillus* spp. (*B. licheniformis* and *B. subtilis*) the most sensitive, followed by *Staphylococcus aureus* and *Micrococcus luteus.* Regarding the antifungal activity, Frs. 1–3 also displayed the best activities, but only against *P. italicum* (GIZ around 9.0 mm), while the crude EO showed no antifungal activity. Overall, the best antimicrobial properties of Frs. 1–3 could be attributed, at least in part, to their highest content in α-pinene and bornyl acetate. On the other hand, Frs. 4–6 (>30 min HDT) exhibited the strongest antioxidant activities (EC_50_ values: 1.5–2.3 and 1.0–1.7 mg mL^−1^ for DPPH and ABTS, respectively), being at least 1.3-fold higher than those of the crude EO. The presence of nezukol, elemol, and eudesmol isomers could strongly contribute to the best free radical scavenging properties of Frs. 4–6. In conclusion, HD was found to be an efficient process for obtaining new Az–CJF EO fractions with variable and enhanced bioactivities due to their differential composition, as assessed using GC–MS. Hence, these findings could contribute to increasing the commercial potential of the *C. japonica* EO industry, namely, the Fr2 and Fr6, which presented the most significant activities and can have potential applications in the food, medical, and agriculture sectors.

## 1. Introduction

Antimicrobial drugs, such as antibiotics and antifungals, are used to treat, prevent, or control infectious diseases in humans, other animals, and plants. However, the emergence of new resistant microbial strains, and re-emergence of known pathogens, to current synthetic antimicrobials, combined with a decline in the development of new antibiotic and antifungal drugs, presents a major problem for both public and animal health in the 21st century. Antimicrobial resistance (AMR) also poses a constant threat to food safety management systems worldwide, with plant diseases recognized as one of the biggest challenges. In fact, several important crops are susceptible to infection by pathogenic bacteria and/or fungi, both in the field and/or during postharvest stages, with a significant impact on foodstuff loss worldwide [1,2,3,4]. Moreover, global regulations concerning pesticide residue levels in fruits and vegetables have become increasingly stricter over the years for human health and environmental protection purposes [5]. In addition, food deterioration, via lipid oxidation during food processing and storage, is another complex problem since most synthetic antioxidants, widely used nowadays, such as butylated hydroxyl toluene (BHT) and butylated hydroxyl anisole (BHA), can also have adverse effects on human health [6]. On the other hand, the growing waste generation is currently a serious issue due to (i) an increase in consumption directly related to the world`s population growth and (ii) the linear system of industrialization that is still under transition into a circular system. Therefore, the reduction of waste through its transformation into value-added products is a global priority [7].

The above-mentioned concerns have resulted in increased attention to bioactive natural products, namely, from plants (including their biomass residues), such as the essential oils (EOs) that have a long history of being used by humans and possess “generally recognized as safe” (GRAS) status, attributed by the Food and Drug Administration (FDA). In fact, during the last decades, the antimicrobial [5,8,9,10,11,12,13,14] and antioxidant [6,11,12,13,14] properties inherent to EOs from various aromatic and medicinal plants have been intensively investigated, evidencing that they may represent an effective eco-friendly alternative for applications in several fields, including food, medical, and agriculture sectors [12,13,14,15]. Such dual antibacterial–antioxidant activity allows for these EOs to have, for example, a high potential as natural food additives because of their preservative properties for enhancing the safety and stability of foodstuffs. It is also noteworthy that the EO antimicrobial activity is attributed to several distinct mechanisms due to their complex bioactive composition, thus preventing the development of AMR [12,13]. Therefore, EOs could be promising therapeutics to combat ESKAPEE pathogens (i.e., *Enterococcus faecium*, *Staphylococcus aureus*, *Klebsiella pneumoniae*, *Acinetobacter baumannii*, *Pseudomonas aeruginosa*, *Enterobacter* spp., and *Escherichia coli*) [10]. Recent studies indicate that the application of encapsulated EOs, which supports their controlled and sustained release, enhances their bioavailability and efficacy against AMR [8].

The EOs are, in general, volatile complex mixtures, mainly constituted by terpenes (including mono-, sesqui-, and diterpenes) and their oxygenated derivatives (terpenoids), which represent the largest and most diverse group of plant secondary metabolites. Typically, the major essential oil components (EOCs) are the main responsible factor for EO bioactivities. Nevertheless, it is difficult to ascribe the bioactive effects (for example, antimicrobial or antioxidant properties) of an EO to one or a few major active components since minor EOCs may also play an important role in EO bioactivity, either by potentiating the action of major EOCs or through antagonistic or additive effects [12,16]. Furthermore, the main biological effect might be coming from minor EOCs [17]. This highlights the importance of the EO fractionation to enhance target compound content. In fact, some authors have previously noted the potential for enhanced biological activities in conifer EOs through fractionation during the distillation process, such as hydrodistillation (HD) [18,19], due to a different chemical structure of the EOCs and, consequently, their differential physicochemical properties, namely, volatility and vapour pressure.

The remarkably increasing applications and markets for EOs could bring new opportunities for the sustainable management of unused forestry biomass residues, such as the ones from Azorean *Cryptomeria japonica* (Thunb. ex L. f.) D. Don (a rich source of valuable EOs), with social, environmental, and economic impact. This conifer species, belonging to the Cupressaceae family, is native to Japan. It is an evergreen tree, up to 70 m high, with a very large trunk (diameter up to 4 m) and spirally arranged leaves, reduced in size (0.5–1 cm long) and needle-like in appearance [20]. *Cryptomeria*, a monotypic genus [20], is among the conifer genera that have an enormous capacity to synthesize a complex terpenoid mixture (oleoresin) mainly stored in well-developed secretory ducts, which acts as a strong defense system, thus contributing to their evolutionary diversification and colonization success [21]. In fact, *C. japonica* is widely cultivated in plantation forests in Asian countries (e.g., Japan, China, Korea, and Taiwan), as well as in the Azores archipelago (Portugal) due to its timber quality, including natural decay resistance [22]. Thus, a huge amount of *C. japonica* biomass residues (CJBR), particularly *C. japonica* foliage (CJF), is generated from the wood industry and forest operations, which can cause several environmental problems. However, these CJBR remain valuable resources that can be used to produce value-added products, such as EOs [15] and plant extracts [23].

CJF EO and their fractions, or EOCs, could find application in different commercial fields (e.g., food, cosmeceutical, pharmaceutical, medical, and agrochemical), with manifold approaches, due to their valuable multi-bioactivities, as determined over the last decades and summarized in a recent critical review [15]. However, it should be highlighted that the specific commercial applications of EOs will depend mainly on the chemical profile, which, in turn, is significantly influenced by several factors, such as: (i) the geographical region of the plants, growing environments (abiotic or biotic factors), and harvest period; (ii) the plant genetic background, age, and developmental stage; (iii) postharvest processing of plant material that involves cleaning, drying, and storage procedures; and (iv) the extraction method and protocol used [24]. As a result, the comparison of data between different studies is very difficult when different vegetable raw materials, processing conditions, extraction protocol, analytical methods, and/or units of measurement are used, among other factors (e.g., experimental conditions of bioassays).

It is also noteworthy that different exogenous and endogenous factors can lead to distinct ecotypes or chemotypes in the same plant species [24]. For example, it was demonstrated by principal component analysis and hierarchical cluster analysis that the CJF EO chemotype is α-pinene type in the Azores, while in most Asian countries, it is either ent-kaurene type or elemol plus ent-kaurene type. Nevertheless, the CJF EO chemotypes from several countries exhibited broad-spectrum antimicrobial activities, among other biocidal properties (e.g., antiviral, acaricidal, molluscicide, mosquito larvicidal, and termiticidal activities), as well as other important pharmacological properties (e.g., antioxidant, anti-inflammatory, anticancer, neuroprotective, anxiolytic, antitussive, antiulcer, antimelanogenesis, and skin whitening) [15], with potential application in integrated pest management programs (IPMP), and/or human and other animals’ health areas.

As part of our continuing phytochemical investigation and bioactivity studies of CJBR and as an incentive for local *C. japonica* EO industry development, it was recently observed that Azorean CJF (Az–CJF) EO obtained via HD exhibits weak to moderate antibacterial activity against selected Gram-positive bacteria [20]. On the other hand, as also reported previously, collecting Az–CJF EO at different HD timeframes (HDTs: 0–2, 2–10, 10–30, 30–60, 60–120, and 120–240 min) during the HD process resulted in EO fractions with distinct yields, physical properties, and chemical compositions, in comparison with crude Az–CJF EO obtained via a typical HD over 4 h. Thus, new potential high value-added products were obtained [25].

In this context, the present study aimed to investigate the aforementioned Az–CJF EO fractions (Frs. 1–6) in regard to their (i) in vitro antimicrobial activities against seven bacteria and two *Penicillium* spp. fungi and (ii) in vitro antioxidant activity, evaluated using two free radical scavenging activity (FRSA) assays, 2,2-diphenyl-1-picrylhydrazyl (DPPH), and 2,2′-azino-bis-3-ethylbenzothiazoline-6-sulfonic acid (ABTS). The obtained typical crude Az–CJF EO was used as a control sample (control EO). In addition, the antimicrobial effects of some pure EOCs (α-pinene, terpinen-4-ol, and bornyl acetate) were determined against the selected microorganisms. It should be noticed that the use of the HD process as a step to obtain EO fractions to perform biological analyses is yet scarce in species of the Cupressaceae family [19,26]. Furthermore, to the best of our knowledge, no prior studies on the EOs from *C. japonica* with the same purpose have been reported so far. Overall, the results of this study can help the *C. japonica* EO industry to produce EO fractions with differential bioactivities, due to their distinct chemical compositions, thus contributing to the potential biovalorization of waste from abundant Azorean resources and, consequently, to the local bioeconomy and circular economy.

## 2. Materials and Methods

### 2.1. Chemicals and Reagents

A standard mixture of C7–C33 *n*-alkanes was obtained from Restek (Bellefonte, PA, USA). (–)-α-Pinene (≥97%), (–)-terpinen-4-ol (≥95%), (–)-bornyl acetate (≥95%), clotrimazole, kanamycin, 6-hydroxy-2,5,7,8-tetramethylchroman-2-carboxylic acid (Trolox), anhydrous sodium sulfate (Na_2_SO_4_), Tween 20, DPPH, ABTS, dimethylsulfoxide (DMSO), and potassium persulfate (K_2_S_2_O_8_) were obtained from Sigma-Aldrich (St. Louis, MO, USA). Nutrient agar, Muller–Hinton agar (MHA), and potato dextrose agar (PDA) were purchased from Merck (Darmstadt, Germany). Methanol (HPLC grade) was obtained from Riedel-de Häen (Aktiengesellschaft, Seelze, Germany).

### 2.2. Plant Material

The CJF were harvested during the pollination stage in early March 2023 (winter season) from a tree population located on Lomba da Maia (latitude 37°48′32.7″ N, longitude 25°20′06.5″ W, altitude 440 m) in the northeast region of São Miguel Island (Azores archipelago, Portugal). The plant material, randomly cut off from healthy plants, was placed in plastic bags and immediately brought to a laboratory at the University of the Azores where the strobili attached to the foliage were removed. The fresh CJF sample was immediately stored at −20 °C until further usage in the HD process. Prior to this process, the sample was cut into small chips, about 2 cm in length.

A voucher specimen was deposited in the Herbarium AZB–Ruy Telles Palhinha of the University of the Azores under number AZB 4581.

### 2.3. EO Isolation and Fractionation by HD

A Clevenger-type apparatus was used to obtain and fractionate the EO from Az–CJF, as reported in Arruda et al. [25]. Briefly, the EO fractions were collected in the following sequential HDTs: 0–2, 2–10, 10–30, 30–60, 60–120, and 120–240 min (Frs. 1–6). In addition, a control EO sample (crude EO) was collected from a non-fractionated HD (0–240 min). The collected fractions and control were dehydrated with anhydrous Na_2_SO_4_ and stored in sealed amber vials at 4 °C until further analysis.

Each HD was performed in triplicate, and the EO yield (%) was calculated as the EO mass (g) per 100 g of fresh weight (f.w.) of CJF.

### 2.4. EO Composition Analysis

The chemical composition of the EOs samples was determined using gas chromatography/mass spectrometry (GC/MS) analysis, using a Shimadzu GCMS–QP2010 ultra gas chromatograph mass spectrometer fitted with a ZB–5MSPlus (5% phenyl, 95% methyl siloxane) capillary column (60 m × 0.25 mm i.d., 0.25 µm film thickness) from Phenomenex Inc. (Torrance, CA, USA). The oven’s temperature was set at 2 °C min^–1^ from 50 °C to 260 °C, being then held at 260 °C for 5 min. The injector, GC/MS interface, and ion source temperatures were set at 260 °C. The transfer line temperature was set at 300 °C. A volume of 0.1 μL of EO sample dissolved in methylene chloride (0.1 g mL^–1^) was injected in the split mode at a ratio of 24.4:1. Helium was used as the carrier gas with a flow rate of 36.3 cm s^−1^. Mass spectra were recorded at 70 eV where the mass scan range was 40–400 atomic mass units (amu) with a scan time of 0.3 s [25]. Retention indices (RI) were calculated according to ISO 7609 [27], relative to a homologous series of *n*-alkanes (C_7_–C_33_).

Identification of the EOCs was performed by comparison of their RI and GC–MS spectra with corresponding data from a lab-made library with commercially available standards and components from reference EOs, as well as from other GC–MS databases (FFNSC4.0, NIST11, and Wiley10) [25,28]. For quantification, the EOC raw percentage was calculated by integrating total ion current (TIC) chromatogram peaks without correction factors as the mean values of three injections from each EO sample [25].

### 2.5. In Vitro Antimicrobial Activity Determination

#### 2.5.1. Microorganisms, Growth Conditions, and Inocula Preparation

The studied microorganisms were obtained from the collection of the Microbiology Laboratory at the University of the Azores. The seven selected bacterial strains (foodborne and/or human pathogens) include (i) four Gram-positive, namely, *Bacillus subtilis* (Ehrenberg) Cohn (DSM 10), *Bacillus licheniformis* (Weigmann) Chester (DSM 13), *Staphylococcus aureus* Rosenbach (DSM 1104), and *Micrococcus luteus* (Schroeter) Cohn (DSM 20030), and (ii) three Gram-negative bacteria, namely, *Serratia marcescens* Bizio (DSM 48), *Escherichia coli* (Migula) Castellani & Chalmers (DSM 498), and *Entereobacter cloacae* (Jordan) Hormaeche & Edwards (DSM 30054). Among the selected bacterial strains, *S. aureus*, *E. cloacae,* and *E. coli* belong to ESKAPEE pathogens, a group of seven highly virulent and AMR bacterial strains [10]. The two selected filamentous fungal isolates (phytopathogens), namely, *Penicillium digitatum* (Person) Saccardo and *P. italicum* Wehmer, were previously obtained from infected citrus fruits with the typical green and blue mold symptoms, respectively [29,30].

The bacterial strains were cultured and maintained on nutrient agar. Bacterial inocula were prepared by the direct inoculation of colonies in sterile saline solution adjusted to a 0.5 McFarland standard.

The fungal strains were maintained on PDA medium at 25 °C for 4 to 5 days (until spore formation). The fungal spore suspensions were prepared by resuspending a 7-day-old pure culture in sterile distilled water, containing 0.5% Tween 20, and adjusting the concentration to 10^5^ spore mL^−1^ using a Neubauer improved hemocytometer (Hirschmann, Eberstadt, Germany).

#### 2.5.2. Disc Diffusion Method (DDM)

The DDM described by Kirby–Bauer [31], with some modifications, was used to evaluate the antimicrobial activity of the EO and EOC samples under study. Briefly, 5 µL of undiluted sample was loaded onto a 6 mm diameter sterile paper disc and placed directly on swab-inoculated MHA and PDA plates for bacteria and fungi, respectively. Special care was taken to ensure uniform contact of the paper disc with the media surface. The microbial cultures were incubated during 24 h at 28 °C and 37 °C for Gram-positive and Gram-negative bacteria, respectively, and during 72 h at 25 °C for fungi. After incubation, the diameters of the growth inhibition zones (GIZ) were measured in mm, including the diameter of the disc. As positive controls, 5 µL of kanamycin and clotrimazole solutions (10 mg mL^–1^ in water and 5 mg mL^–1^ in DMSO, respectively) were used in antibacterial and antifungal assays, respectively. Discs with sterile water were used as negative control and inoculated plates without samples were used as growth controls in both assays. All assays were performed in triplicate.

### 2.6. In Vitro Antioxidant Activity Evaluation by Free Radical Scavenging Activity (FRSA) Assays

The FRSA of the EO samples, with various concentrations (range of 0.15–150 mg mL^−1^), and Trolox (positive control sample) were determined by measuring their abilities to quench the DPPH stable free radical and the ABTS radical cation, according to Blois [32] and Re et al. [33] methods, respectively, with some modifications. In both assays, a mixture without an EO sample or Trolox was used as the control. The assays were performed using a 96-well plate, with the absorbance (Abs) measured using a microplate reader (Thermo Scientific Multiskan FC, Waltham, MA, USA).

For DPPH–FRSA assay, a 100 µL aliquot of each sample was mixed with 100 µL of DPPH solution (0.08 mg mL^−1^ in methanol). The plate was shaken and incubated in the dark for 30 min at room temperature, and then Abs was measured at 520 nm.

For the ABTS–FRSA assay, the ABTS radicals were obtained by reacting 7 mmol L^−1^ ABTS with 2.45 mmol L^−1^ K_2_S_2_O_8_ and allowing the mixture to stand in the dark at room temperature for 16 h. Afterward, the ABTS solution was diluted with methanol until an Abs of 0.7 was reached at 734 nm. Then, a 100 µL aliquot of each sample was added to 100 µL of ABTS solution. The plate was shaken and incubated in the dark for 6 min at room temperature, and then Abs was measured at 734 nm.

The FRSA was calculated as a percentage of radical (DPPH or ABTS) discoloration using the following Equation (1):

(1) $$FRSA\left(\%\right)=1-\left(\frac{{Abs}_{sample}}{{Abs}_{control}}\right)\times 100$$

The results are expressed as a half-maximal effective concentration (EC_50_) value (mg mL^−1^), which is defined as the sample concentration needed to quench fifty percent of the DPPH or ABTS free radical quantity. A lower EC_50_ value means a higher antioxidant activity. All measurements were performed in triplicate.

### 2.7. Statistical Analysis

All experiments were performed in three replicates, and data are expressed as the mean ± standard deviation (SD). The normal distribution of variables was tested with a Shapiro–Wilk test, and when this assumption was not met, data were transformed prior to using the ANOVA procedure. Significant differences between groups were calculated using Duncan’s multiple-range test at a 5% significance level (*p* < 0.05). All analyses were conducted using IBM SPSS Statistics version 28.0.1.0 software (SPSS Inc., Chicago, IL, USA).

## 3. Results and Discussion

### 3.1. Chemical Composition and Yield of the Az–CJF EO and Its Fractions

**Figure 1:**
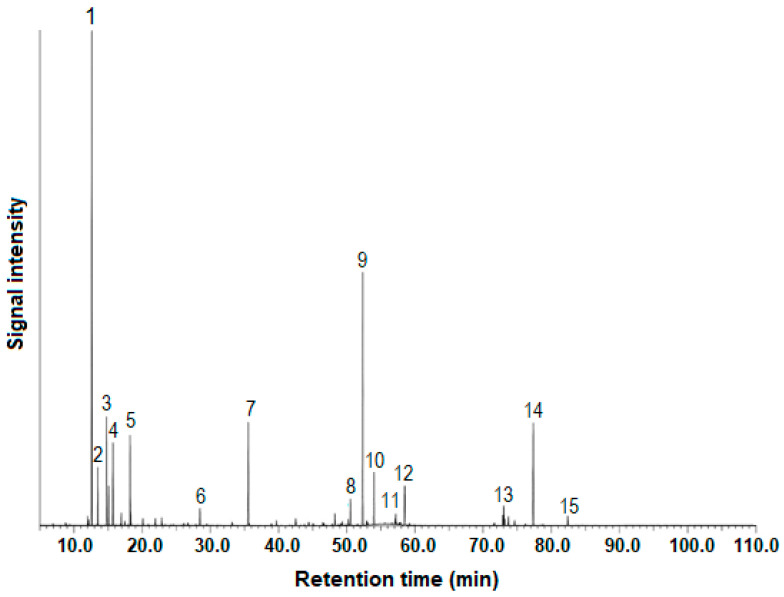
Total ion current (TIC) chromatogram on a ZB–5MSPlus column of the fraction 2 (2–10 min) from Azorean *Cryptomeria japonica* foliage essential oil obtained via hydrodistillation. For components name of peaks 1–15, please see Table 1.

As already highlighted, the EO bioactivities, including antimicrobial or antioxidant, would be expected to relate to their chemical composition and synergistic interactions between active EOCs [8]. Thus, the concentration of certain molecules, which can be achieved by EO fractionation during the HD process [25], is often more important than complete purification. Table 1 summarizes the yield of the studied EO samples, as well as their chemical composition, namely, (1) the major EOCs (≥1%), (2) the percentage of the grouped EOCs: monoterpene hydrocarbons (MH), oxygen-containing monoterpenes (OCM), sesquiterpene hydrocarbons (SH), oxygen-containing sesquiterpenes (OCS), diterpene hydrocarbons (DH), and oxygen-containing diterpenes (OCD), and (3) the terpenes/terpenoids ratio values. In addition, Figure 1 illustrates the chromatographic profile of a representative Az–CJF EO fraction.

Overall, MH prevailed in Fr1 and Fr2 (92 and 45%, respectively, mainly α-pinene) while OCS dominated in Frs. 3–6 and control EO (42–62%, mainly elemol and eudesmol isomers). A yield variation is also observed within the studied Az–CJF EO fractions.

### 3.2. In Vitro Biological Activities of the Az–CJF EO and Its Fractions

#### 3.2.1. Antibacterial Activity

The antibacterial activity of the Az–CJF EO samples collected at different HDTs, as well as of the selected EOCs (α-pinene, terpinen-4-ol, and bornyl acetate) and the kanamycin drug, was evaluated by the DDM, and the results, expressed as GIZ, are presented in Table 2.

As shown in Table 2, all studied EO samples (Az–CJF EO and its six fractions) showed antibacterial activity against all tested Gram-positive bacteria (GIZ of 7.0–23.3 mm), except for the Fr1, which was inactive toward *M. luteus*. Contrariwise, it has been shown that all studied EO samples were inactive against all tested Gram-negative bacteria. A similar trend has already been observed for other Azorean *C. japonica* plant parts [20] and in other conifers, such as the foliage EO from *Abies balsamea* L. Mill. (Pinaceae) [34]. Generally, Gram-positive bacteria are more susceptible to EOs than Gram-negative bacteria. This is likely due to the thick peptidoglycan layer and absence of an outer membrane in Gram-positive bacteria, which allows for easier access to antibacterial molecules. Conversely, Gram-negative bacteria have an outer membrane composed of a double layer of phospholipids linked to the inner membrane by lipopolysaccharides, limiting the diffusion of hydrophobic compounds and reducing EO efficacy [35]. However, some studies have not found Gram-positive bacteria to be more susceptible to EOs [10]. Concerning the antibacterial activity of the tested EOCs (Table 2), it was observed that α-pinene, the major MH of all studied EO samples (Table 1), also exhibited, in general, superior efficacy against Gram-positive bacteria over negative ones (GIZ of 9.0–17.0 mm and 8.0–10.0 mm, respectively), while bornyl acetate, the major OCM (Table 1), was only active against the tested Gram-positive bacteria (GIZ of 8.0–21.0 mm), except for *M. luteus*. On the other hand, terpinen-4-ol, the second major OCM (Table 1), was effective against both Gram-positive and Gram-negative bacteria (GIZ of 7.3–22.0 mm and 17.7–30.7 mm, respectively), revealing a remarkable antibacterial activity against all tested Gram-negative bacteria.

Notably, Fr2 showed significantly higher activity (GIZ of 14.3–23.3 mm), which, compared with that of the control EO (GIZ of 8.3–11.7 mm), represents a 1.4- to 2.2-fold improvement in the Az–CJF EO effectiveness as an antibacterial agent. The Fr3 was the second-best antibacterial agent (GIZ of 9.0–13.3 mm), although not significantly different from the control EO.

*Bacillus* spp. were found to be, in general, more sensitive to the studied EO samples than *S. aureus* and *M. luteus* species. Among *Bacillus* spp., the most susceptible was *B. licheniformis* (GIZ of 10.0–23.3 mm), a bacterium that survives pasteurization and other heat treatments as spores; thus, toxin-producing strains of this species in the dairy production chain may be of food safety concern [36,37]. Frs. 1–3 exhibited significantly higher activities against this pathogen (GIZ of 13.3, 23.3, and 13.3 mm), which, compared with that of the control EO (GIZ of 11.7 mm), represent a 1.14- to 2.0-fold improvement.

Regarding the composition of all Az–CJF EO samples under study, the data presented in Table 1 revealed that the referred fractions (Frs. 1–3) showed the highest MH content, mainly α-pinene (54, 24, and 13%, respectively) and the highest terpenes/terpenoids ratio values. In addition, a similar pattern was observed in Fr2 and Fr3, which were the richest in OCM and SH content. Among the OCM components of Fr2 and Fr3, bornyl acetate accounted for 6.3% and 3.9%, respectively, representing a 3.7- and a 2.3-fold increase in content compared with the control EO, respectively. Similarly, the amount of terpinen-4-ol in Fr3 was significantly higher than that in the control EO. Concerning the SH components of Fr2 and Fr3, δ-cadinene accounted for 2.0% and 2.6%, respectively, representing a 1.9- and a 2.4-fold increase in content compared with the control EO, respectively.

Interestingly, it could be observed that Fr2 (the most active antibacterial sample as already highlighted) showed higher activity against the two *Bacillus* spp. than that of α-pinene (Table 2), its major component (Table 1), which showed weak to moderate activity against these bacteria. This suggests the role of other EOCs, such as the terpenoid compounds that, in general, exhibit better antimicrobial activity than the hydrocarbon congeners [38]. Indeed, the OCM bornyl acetate showed strong antibacterial activity against the two *Bacillus* spp. (Table 2). Bornyl acetate is a key component in several proprietary Chinese medicines and is found in EOs from Cupressaceae plants. It has notable pharmacological activities, including anticancer, anti-inflammatory, and antimicrobial properties [38,39,40]. Additionally, due to its surfactant nature, bornyl acetate is an effective sporicidal agent, particularly against *B. subtilis* and other spore-forming bacteria, which are resistant to heat, physical, and chemical treatments, making them difficult to control in the food industry [41]. Thus, future studies should consider Az–CJF EO Frs. 1–3 (particularly Fr2, the richest one in bornyl acetate) for further investigation on their potential sporicidal activities against *Bacillus* spp. and other spore-forming bacteria.

Based on the results obtained in this study, and published data availability [34], the antibacterial activity of Fr2 could be due to its distinct active chemical profile, as characterized above. Moreover, a synergistic effect of its minor components, such as the SH β-caryophyllene and α-humulene [25], could contribute to the observed higher activity.

Finally, it was observed that all Gram-positive bacteria were sensitive to α-pinene (Table 2), but Fr1, which was the richest in this MH (Table 1), was inactive toward *M. luteus* and weakly active toward *S. aureus* (Table 2). This could be due to antagonistic interactions amongst α-pinene and other Fr1 EOCs as a result of different (+) and (−) enantiomer ratios of α-pinene in Az–CJF EO Frs. 1–3. In fact, it is reported that α-pinene with different enantiomeric compositions in EOs from *Juniperus communis* L. has diverse antimicrobial effectivities toward microorganisms [42]. Therefore, it is very important to determine the α-pinene enantiomeric composition of EO Frs. 1–3, which should be considered for further investigation.

Overall, this study clearly confirms that the use of HD to fractionate Az–CJF EO significantly increased their growth-inhibitory activity against Gram-positive bacteria, possibly due to enhancing target compound content, but had no effect against the selected Gram-negative bacteria. Specifically, Frs. 1–3 (<30 min HDT) have, in general, the best inhibitory effects on bacterial growth, which could be attributed, at least in part, to their highest content of α-pinene and bornyl acetate. Among these fractions, the most effective was Fr2 (2–10 min HDT), having presented strong antibacterial activity against *B. licheniformis*, *B. subtilis,* and *S. aureus* and moderate to strong activity against *M. luteus*. It is also noteworthy, however, that despite possessing the highest OCM content, a factor that could create the expectation of Fr2 inhibiting Gram-negative bacteria development (due to the usually observed efficacy of OCM against these organisms), it was revealed that this fraction did not show antibacterial activity against this type of bacteria, possibly due to the insufficient OCM content in Fr2 to properly display such activity.

#### 3.2.2. Antifungal Activity

Similarly, the antifungal activity against *P. digitatum* and *P. italicum* of the studied EO samples, as well as of the selected EOCs (α-pinene, terpinen-4-ol, and bornyl acetate) and the clotrimazole drug, was also evaluated by the DDM, and the results, expressed as GIZ, are presented in Table 3. These fungi were selected because they are the most economically significant pathogens in citrus, causing substantial postharvest losses of up to 30% and 80%, respectively, and reducing the product’s shelf life [3].

As shown in Table 3, all studied EO samples had no antifungal activity against *P. digitatum*. On the other hand, only Frs. 1–4 displayed antifungal activities against *P. italicum* despite weak (GIZ of 7.3–9.3 mm) and showing no statistical differences between them. Interestingly, a similar antifungal activity against *P. italicum* (GIZ of 9 mm) was exhibited by the EO obtained via HD of the immature female cones removed from the CJF sample under study [43].

**Figure 2:**
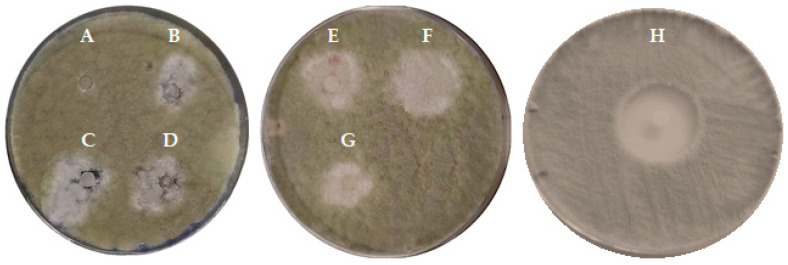
Inhibition of fungal sporulation in *Penicillium digitatum*. Legend: A—Fr1, B—Fr2, C—Fr3, D—Fr4, E—Fr5, F—Fr6, G—Control EO and H—Clotrimazole.

However, it is important to highlight that although no GIZ were observed for *P. digitatum* (Table 3 and Figure 2), it was noted that all tested EO samples, except for Fr1, exhibited sporulation inhibition characterized by white halo zones (Figure 2). Further studies should explore the fungal sporulation inhibition properties of the Az–CJF EO samples.

As also shown in Table 3, it is noteworthy that the EO samples that demonstrated slightly superior antifungal activities were Frs. 1–3, with the most effective being Fr2, followed by Fr3, a pattern comparable with that reported for the antibacterial activity in Section 3.2.1. As already highlighted, Fr2 and Fr3 were the richest in OCM—namely, terpinen-4-ol and bornyl acetate, the major OCM compounds (Table 1). Among the tested EOCs (Table 3), it was observed that terpinen-4-ol exhibited superior efficacy against *P. italicum* compared with *P. digitatum* (GIZ of 12.7 mm and 9.3 mm, respectively). Similarly, bornyl acetate had weak antifungal activity against *P. italicum* (GIZ of 7.3 mm) but no effect on the *P. digitatum* growth inhibition, while α-pinene, one of the major EOCs of the studied Az–CJF EO samples (Table 1), presented no activity against both fungal strains.

Scora and Scora [44] evaluated the effect of many EOCs on mycelium growth of *P. digitatum*, *P. italicum,* and *P. ulaiense* Hsieh, Su & Tzean. They reported that terpinen-4-ol, followed by bornyl acetate, presented higher activity against *P. italicum*, compared with α-pinene, which is in good agreement with our results on Az–CJF EO antifungal activity. Furthermore, Scora and Scora [44] found that the fungicidal activity of the tested EOCs varied among the three fungi, with *P. digitatum* being the least sensitive and *P. ulaince* being the most sensitive. Again, this finding aligns with the present study where *P. digitatum* was also found to be the least sensitive. Indeed, *P. digitatum* is recognized as a *Penicillium* spp. with higher resistance to antifungal agents, including terpenes, attributed to their ability to convert certain terpenes into less harmful compounds via mycelia and spores. Consequently, this elucidates why *C. japonica* EO samples exhibit greater efficacy against *P. italicum* mycelial growth compared with *P. digitatum* [45].

Another study [46] showed that a fraction of elemol + eudesmol isomers (α and γ), obtained via chromatography of CJF EO from South Korea, demonstrated a significant antifungal effect against *Trichophyton rubrum* (Castell.) Sabour, the most common dermatophyte fungus. In the present study, Frs. 3–6 were the richest in elemol and eudesmol isomers (α and γ) (Table 1). Although these fractions did not show favorable results against *P. digitatum* and *P. italicum*, they could be effective in inhibiting the growth and development of other pathogenic fungi, such as dermatophytes, which should be considered for further investigation.

Overall, this study clearly confirms that the use of HD to fractionate Az–CJF EO had a positive impact on the *P. italicum* growth inhibition, possibly due to the increase in antifungal compounds (such as OCM) in Frs. 1–4, but had no effect against the *P. digitatum* mycelial growth.

#### 3.2.3. Antioxidant Activities Evaluated by DPPH and ABTS Assays

Table 4 shows the FRSA (DPPH and ABTS) of the Az–CJF EO samples collected at different HDTs, as well as of Trolox (positive control), expressed as the half maximal effective concentration (EC_50_) value.

The results show that the FRSA of the studied EO samples decreased as follows: Fr6 > Fr5 ≈ Fr4 > control EO > Fr3 ≫ Fr2 > Fr1 for DPPH and Fr6 > Fr5 > Fr4 > Fr3 > control EO > Fr2 ≫ Fr1 for ABTS assays. Thus, the results revealed that the DPPH and ABTS scavenging activities followed a similar pattern in the EO samples under study, which is explained by the fact that both assays rely on a mechanism of electron/hydrogen donation. However, all studied EO samples presented better ABTS than DPPH scavenging activities (EC_50_ values of 1.01–22.31 and 1.48–49.01 mg mL^−1^, respectively), particularly for the early fractions (Table 4).

Concerning the chemical composition of the Az–CJF EO samples under study (Table 1), it was observed that, in general, a similar chemical profile was observed in the control and Frs. 4–6 EO samples, characterized by higher OCS (50–62%, mainly elemol, followed by α+β+γ eudesmol isomers), DH (18–23%, mainly phyllocladene), and OCD (2–4%, mainly nezukol) contents. Furthermore, the aforementioned EO samples display similar terpenes/terpenoids ratio values, namely, 0.71, 0.73, 0.71, and 0.4 for control, Fr4, Fr5, and Fr6, respectively, which were the lowest ratios among all studied EO samples. According to the published data availability, some major EOCs, such as the hydroxylated sesquiterpenes elemol, α-eudesmol, and mainly γ-eudesmol are potent single electron transfer-based agents [47], which explains, at least in part, why the later Az–CJF EO fractions exhibited higher antioxidant activity.

Furthermore, a previous study [48] on the antioxidant activity of CJF EO from South Korea highlighted the strong contribution of nezukol (a hydroxylated tricyclic diterpene), as a radical scavenger or primary antioxidant. Nezukol, a not very well-known OCD compound, is found in conifer species’ leaves and heartwood [48], but more recently, it has been reported to occur in *Isodon rubescens* (Hemsley) H. Hara (Lamiaceae), a medicinal plant native to Eastern China [49].

In the present study, as shown in Table 1, the OCD and nezukol contents of the studied EO samples decreased as follows: Fr6 > Fr5 ≈ control EO > Fr4 > Fr3 > Fr2 ≫ Fr1. In general, this order is in accordance with that reported for the antioxidant effectiveness of the studied EO samples. In addition, a similar trend was observed for the OCS content, which decreased as follows: Fr6 > control EO ≈ Fr5 ≈ Fr4 > Fr3 ≫ Fr2 ≫ Fr1. Thus, among the studied EO samples, the most effective antioxidant agent was Fr6 due to its distinct composition characterized by the lowest terpenes/terpenoids ratio value. This finding is in good agreement with other authors, such as Amorati et al. [50], who stated that the EO antioxidant activity is closely related to the presence of terpenoids.

Overall, this study confirms that the use of HD to fractionate Az–CJF EO had a positive impact on the FRSA. In fact, the DPPH and ABTS scavenging activities of Frs. 4–6 (>30 min HDT) were 1.3–2.0 and 1.3–2.2 times higher than that of the control EO (Table 4), with the most effective being Fr6 (120–240 min HDT). The presence of nezukol, elemol, and eudesmol isomers in these fractions could strongly contribute to their highest free radical scavenging properties.

#### 3.2.4. Dual Antimicrobial–Antioxidant Activity

Regarding all determined biological activities, Fr2 (2–10 min HDT) appears as the most promising source of multi-bioactivities among the studied Az–CJF EO fractions. In fact, compared with the crude EO, Fr2 presented remarkable antibacterial activity against selected Gram-positive bacteria, namely, *B. licheniformis*, *B. subtilis*, *S. aureus*, and *M. luteus*, known for their pathogenicity [51,52,53] and/or increasing bacterial resistance to antibiotics [54]. Thus, Fr2 shows potential for commercial applications as an anti-Gram-positive bacterial agent.

In addition, Fr2 presented the highest GIZ value for *P. italicum* (one of the main responsible pathogens for postharvest diseases in oranges [55]); however, the crude EO had no antifungal effectiveness. Furthermore, Fr2 presented a relatively high ABTS scavenging activity even though it was lower than that of the crude EO (EC_50_ values of 3.49 and 2.25 mg mL^−1^, respectively).

However, it should also be noted that concerning the yield of the Az–CJF EO fractions under study (Table 1), the lowest values were found, precisely, in Fr2 (0.061%), which represents 9% of the total extracted EO. Further ongoing studies will involve grinding the Az–CJF sample in order to enhance the EO extraction efficiency, compared with the current process of cutting samples into small pieces.

## 4. Conclusions

The results from this research clearly validated our hypothesis that fractionating Az–CJF EO during the HD process is a valuable tool for obtaining EO fractions with enhanced antibacterial, antifungal, and/or antioxidant activities, due to its distinct chemical compositions. These novel Az–CJF EO fractions, after an in-depth in vivo and toxicology evaluation, could be used for diverse commercial applications, thereby adding more value to the *C. japonica* EO industry. This, in turn, will contribute to the valorization of fresh CJF waste and promote the local sustainable circular bioeconomy.

However, the chemical instability, hydrophobicity, and volatility of EOs pose a challenge for many of their potential applications. Thus, ongoing studies will involve the microencapsulation of Fr2 and Fr6 to evaluate the effect of different commonly used coating materials on the chemical composition and bioactivities of these EO fractions. In addition, further research should explore the new EO fraction’s activity against a broader number of microorganisms and antioxidant properties.

## Figures and Tables

**Table 1:** Major components (≥1%) and yield of the fractions (Fr) and control from Azorean *Cryptomeria japonica* foliage essential oil (EO) obtained via hydrodistillation (adapted from [25]).

N.	Component	Class	RT	RI	Relative Content (%)						
					Control	Fr1	Fr2	Fr3	Fr4	Fr5	Fr6
**1**	α-Pinene	MH	12.6	928	11.64 ^cd^	54.18 ^a^	24.12 ^b^	12.48 ^c^	8.48 ^e^	10.15 ^de^	6.35 ^f^
**2**	Camphene	MH	13.5	944	1.07 ^d^	4.99 ^a^	2.60 ^d^	1.58 ^c^	1.07 ^d^	1.17 ^d^	0.60 ^e^
**3**	Sabinene	MH	14.8	967	1.77 ^c^	10.25 ^a^	5.16 ^b^	2.29 ^c^	1.01 ^d^	0.79 ^de^	0.26 ^e^
**4**	Myrcene	MH	15.7	983	1.61 ^c^	7.45 ^a^	3.88 ^b^	2.07 ^c^	1.32 ^cd^	1.45 ^cd^	0.78 ^d^
**5**	Limonene	MH	18.2	1024	1.74 ^d^	7.16 ^a^	4.61 ^b^	2.52 ^c^	1.52 ^d^	1.57 ^d^	0.82 ^e^
**6**	Terpinen-4-ol	OCM	28.5	1175	0.65 ^c^	0.17 ^d^	0.97 ^bc^	1.40 ^a^	1.16 ^ab^	0.98 ^b^	0.64 ^c^
**7**	Bornyl acetate	OCM	35.5	1277	1.72 ^d^	2.32 ^c^	6.32 ^a^	3.92 ^b^	2.11 ^c^	1.48 ^d^	0.86 ^e^
**8**	δ-Cadinene	SH	50.2	1510	1.07 ^c^	0.23 ^e^	1.98 ^b^	2.62 ^a^	1.71 ^b^	0.86 ^c^	0.54 ^d^
**9**	Elemol	OCS	52.3	1541	27.47 ^abc^	2.11 ^e^	18.10 ^d^	28.55 ^ab^	30.07 ^a^	25.62 ^bc^	24.78 ^c^
**10**	Germacrene-D-4-ol	OCS	54.0	1568	0.97 ^c^	0.70 ^c^	3.61 ^a^	1.43 ^b^	0.18 ^d^	0.03 ^d^	0.00 ^d^
**11**	γ-Eudesmol	OCS	57.2	1623	6.55 ^b^	0.05 ^e^	0.76 ^e^	2.14 ^d^	4.38 ^c^	6.73 ^b^	11.48 ^a^
**12**	α+β-Eudesmol	OCS	58.5	1646	13.10 ^bc^	0.38 ^f^	3.86 ^e^	8.05 ^d^	11.99 ^c^	15.14 ^b^	21.64 ^a^
**13**	Rosa-5,15-diene	DH	73.0	1921	2.11 ^b^	0.10 ^d^	1.43 ^c^	2.39 ^ab^	2.73 ^a^	2.26 ^b^	1.66 ^c^
**14**	Phyllocladene	DH	77.3	2010	14.00 ^ab^	0.58 ^d^	7.21 ^c^	12.94 ^b^	16.42 ^a^	16.12 ^a^	14.20 ^ab^
**15**	Nezukol	OCD	82.4	2119	2.59 ^b^	0.03 ^f^	0.66 ^e^	1.39 ^d^	2.12 ^c^	2.75 ^b^	3.47 ^a^
Total identified components					97.39	99.75	98.22	97.27	97.04	97.15	97.43
Total monoterpene hydrocarbons (MH)					19.68 ^d^	91.79 ^a^	45.23 ^b^	23.92 ^c^	15.47 ^d^	17.48 ^d^	10.09 ^e^
Total oxygen-containing monoterpenes (OCM)					3.01 ^d^	2.96 ^d^	8.98 ^a^	6.55 ^b^	4.00 ^c^	2.95 ^d^	1.86 ^e^
Total sesquiterpene hydrocarbons (SH)					2.03 ^c^	0.93 ^e^	5.51 ^a^	4.86 ^a^	2.83 ^b^	1.43 ^d^	1.01 ^e^
Total oxygen-containing sesquiterpenes (OCS)					51.12 ^b^	3.28 ^e^	27.44 ^d^	42.17 ^c^	49.73 ^b^	50.85 ^b^	62.39 ^a^
Total diterpene hydrocarbons (DH)					18.65 ^bc^	0.76 ^e^	10.38 ^d^	18.33 ^c^	22.78 ^a^	21.49 ^ab^	18.31 ^bc^
Total oxygen-containing diterpenes (OCD)					2.90 ^b^	0.03 ^f^	0.68 ^e^	1.44 ^d^	2.23 ^c^	2.95 ^b^	3.77 ^a^
Total terpenes					40.36	93.48	61.12	47.11	41.08	40.4	29.41
Total terpenoids					57.03	6.27	37.1	50.16	55.96	56.75	68.02
Ratio terpenes/terpenoids					0.71	15.00	1.65	0.94	0.73	0.71	0.40
**EO yield (%, *w*/*w*, fresh weight)**					0.820 ^a^	0.139 ^c^	0.061 ^e^	0.074 ^de^	0.090 ^d^	0.143 ^c^	0.180 ^b^

Values are mean of *n* = 3. Different superscript letters in the same row indicate significant statistical differences (*p* < 0.05). Legend: RT and RI—retention time (min) and retention indices on a ZB–5MSPlus column, respectively.

**Table 2:** Antibacterial activity (growth inhibition zone) of the fractions and control (C) from Azorean *Cryptomeria japonica* foliage essential oil (EO) obtained via hydrodistillation and some of their components.

EO (HDTs) and Compounds	Growth Inhibition Zone (mm)							
	Gram-Positive Bacteria					Gram-Negative Bacteria		
	*Bacillus* *subtilis*	*Bacillus* *licheniformis*	*Staphylococcus* *aureus*	*Micrococcus* *luteus*		*Escherichia* *coli*	*Enterobacter* *cloacae*	*Serratia* *marcescens*
Fr1 (0–2 min)	10.7 ± 0.9 ^cd^	13.3 ± 2.1 ^c^	7.0 ± 0.0 ^e^	na		na	na	na
Fr2 (2–10 min)	18.0 ± 2.7 ^b^	23.3 ± 1.5 ^a^	15.3 ± 0.4 ^b^	14.3 ± 0.4 ^b^		na	na	na
Fr3 (10–30 min)	10.0 ± 0.7 ^cde^	13.3 ± 1.2 ^c^	10.3 ± 0.4 ^c^	9.0 ± 1.3 ^cd^		na	na	na
Fr4 (30–60 min)	9.3 ± 1.8 ^cde^	11.7 ± 0.6 ^cd^	8.0 ± 0.0 ^d^	9.0 ± 1.3 ^cd^		na	na	na
Fr5 (60–120 min)	7.7 ± 0.4 ^e^	10.7 ± 1.2 ^cd^	8.0 ± 0.0 ^d^	8.5 ±1.5 ^cd^		na	na	na
Fr6 (120–240 min)	7.7 ± 0.4 ^e^	10.0 ± 2.0 ^d^	8.7 ± 0.4 ^d^	9.0 ± 1.3 ^cd^		na	na	na
C (0–240 min)	8.3 ± 0.9 ^de^	11.7 ± 2.1 ^cd^	10.7 ± 0.4 ^c^	10.0 ± 1.0 ^c^		na	na	na
(–)-α-Pinene	9.0 ± 1.0 ^cde^	12.8 ± 1.0 ^c^	17.0 ± 1.0 ^a^	15.7 ± 3.2 ^a^		9.0 ± 1.0 ^b^	10.0 ± 1.0 ^b^	8.0 ± 1.0 ^b^
(–)-Terpinen-4-ol	12.3 ± 0.5 ^c^	22.0 ± 1.0 ^a^	10.3 ± 0.6 ^c^	7.3 ± 0.6 ^d^		17.7 ± 0.6 ^a^	30.7 ± 3.0 ^a^	21.1 ± 1.7 ^a^
(–)-Bornyl acetate	21.0 ± 1.0 ^a^	17.0 ± 3.0 ^b^	8.0 ± 0.0 ^d^	na		na	na	na
Kanamycin	39.0 ± 2.0	34.0 ± 2.0	29.3 ± 2.2	27.3 ± 4.0		26.5 ± 3.0	28.0 ± 7.0	24.0 ± 2.0

Values are mean ± SD (*n* = 3). Different superscript letters in the same column of the same strain indicate significant statistical differences (*p* < 0.05). Legend: HDTs—hydrodistillation timeframes; na—no activity; 7–10 mm (weak activity); 10–15 mm (moderate activity); >15 mm (strong activity).

**Table 3:** Antifungal activity (growth inhibition zone, GIZ) of the control (C) and fractions of Azorean *Cryptomeria japonica* foliage essential oil (EO) obtained via hydrodistillation and some of their components.

EO (HDTs) and Compounds	GIZ (mm) on *Penicillium* spp.	
	*P. digitatum*	*P. italicum*
Fr1 (0–2 min)	na	8.7 ± 1.2 ^b^
Fr2 (2–10 min)	na	9.3 ± 2.5 ^b^
Fr3 (10–30 min)	na	9.0 ± 1.0 ^b^
Fr4 (30–60 min)	na	7.3 ± 0.6 ^b^
Fr5 (60–120 min)	na	na
Fr6 (120–240 min)	na	na
C (0–240 min)	na	na
(–)-α-Pinene	na	na
(–)-Terpinen-4-ol	9.3 ± 0.6	12.7 ± 1.2 ^a^
(–)-Bornyl acetate	na	7.3 ± 0.6 ^b^
Clotrimazole	15.0 ± 0.0	19.7 ± 1.2

Values are mean ± SD (*n* = 3). Different superscript letters in the same column of the same strain indicate significant statistical differences (*p* < 0.05). Legend: HDTs—hydrodistillation timeframes; na—no activity; 7–10 mm (weak activity); 10–15 mm (moderate activity); >15 mm (strong activity).

**Table 4:** DPPH and ABTS free radical scavenging activity (FRSA) of the control and fractions of Azorean *Cryptomeria japonica* foliage essential oil obtained via hydrodistillation.

Sample	HDTs (min)	FRSA (EC_50_, mg mL^−1^)	
		DPPH	ABTS
Fr1	0–2	49.01 ± 11.00 ^f^	22.31 ± 1.19 ^g^
Fr2	2–10	32.57 ± 0.45 ^e^	3.49 ± 0.17 ^f^
Fr3	10–30	5.10 ± 0.34 ^d^	2.02 ± 0.09 ^d^
Fr4	30–60	2.28 ± 0.14 ^b^	1.73 ± 0.10 ^c^
Fr5	60–120	2.14 ± 0.12 ^b^	1.42 ± 0.05 ^b^
Fr6	120–240	1.48 ± 0.11 ^a^	1.01 ± 0.03 ^a^
Control EO	0–240	2.98 ± 0.08 ^c^	2.25 ± 0.19 ^e^
Trolox	-	0.0036 ± 0.0003	0.0049 ± 0.0009

Values are mean ± SD (*n* = 3). Different superscript letters in the same column indicate significant statistical differences (*p* < 0.05). Legend: HDTs—hydrodistillation timeframes; DPPH—2,2-diphenyl-1-picrylhydrazyl; ABTS—2,2′-azino-bis-3-ethylbenzothiazoline-6-sulphonic acid.

## Data Availability

Data are contained within the article.

## Abbreviations

Abs, absorbance; ABTS, 2,2′-azino-bis-3-ethylbenzothiazoline-6-sulphonic acid; AMR, antimicrobial resistance; Az–CJF, Azorean *Cryptomeria japonica* foliage; BHA, butylated hydroxyl anisole; BHT, butylated hydroxyl toluene; CJBR, *Cryptomeria japonica* biomass residues; CJF, *Cryptomeria japonica* foliage; DDM, disc diffusion method; DH, diterpene hydrocarbons; DPPH, 2,2-diphenyl-1-picrylhydrazyl; EC_50_, half maximal effective concentration; EO, essential oil; EOC, essential oil component; Fr, fraction; FRSA, free radical scavenging activity; f.w., fresh weight; GC/MS, Gas chromatography/mass spectroscopy; GIZ, growth inhibition zones; HD, hydrodistillation; HDT, hydrodistillation timeframe; MH, monoterpene hydrocarbons; MHA, Muller–Hinton Agar; OCD, oxygen-containing diterpenes; OCM, oxygen-containing monoterpenes; OCS, oxygen-containing sesquiterpenes; PDA, potato dextrose agar; RI, retention indices; SH, sesquiterpene hydrocarbons; TIC, total ion current.
